# Melanoma-Derived *BRAF^V600E^* Mutation in Peritumoral Stromal Cells: Implications for *in Vivo* Cell Fusion

**DOI:** 10.3390/ijms17060980

**Published:** 2016-06-21

**Authors:** Zsuzsanna Kurgyis, Lajos V. Kemény, Tünde Buknicz, Gergely Groma, Judit Oláh, Ádám Jakab, Hilda Polyánka, Kurt Zänker, Thomas Dittmar, Lajos Kemény, István B. Németh

**Affiliations:** 1 Department of Dermatology and Allergology, University of Szeged, Szeged 6720, Hungary; kurgyis.zsuzsanna@med.u-szeged.hu (Z.K.); kemeny.lajos@gmail.com (L.V.K.); buknicz.tunde@med.u-szeged.hu (T.B.); lazarne.olah.judit@med.u-szeged.hu (J.O.); yakoo@freemail.hu (A.J.); 2 MTA-SZTE Dermatological Research Group, Szeged 6720, Hungary; groma.gergo@gmail.com (G.G.); hilda@mail.derma.szote.u-szeged.hu (H.P.); 3 Institute of Immunology & Experimental Oncology, Witten/Herdecke University, Witten 58453, Germany; kurt.zaenker@uni-wh.de (K.Z.); thomas.dittmar@uni-wh.de (T.D.)

**Keywords:** cell fusion, melanoma, mutation detection, macrophage, fibroblast, BRAF^V600E^

## Abstract

Melanoma often recurs in patients after the removal of the primary tumor, suggesting the presence of recurrent tumor-initiating cells that are undetectable using standard diagnostic methods. As cell fusion has been implicated to facilitate the alteration of a cell’s phenotype, we hypothesized that cells in the peritumoral stroma having a stromal phenotype that initiate recurrent tumors might originate from the fusion of tumor and stromal cells. Here, we show that in patients with *BRAF^V600E^* melanoma, melanoma antigen recognized by T-cells (MART1)-negative peritumoral stromal cells express BRAF^V600E^ protein. To confirm the presence of the oncogene at the genetic level, peritumoral stromal cells were microdissected and screened for the presence of *BRAF^V600E^* with a mutation-specific polymerase chain reaction. Interestingly, cells carrying the *BRAF^V600E^* mutation were not only found among cells surrounding the primary tumor but were also present in the stroma of melanoma metastases as well as in a histologically tumor-free re-excision sample from a patient who subsequently developed a local recurrence. We did not detect any *BRAF^V600E^* mutation or protein in the peritumoral stroma of *BRAF^WT^* melanoma. Therefore, our results suggest that peritumoral stromal cells contain melanoma-derived oncogenic information, potentially as a result of cell fusion. These hybrid cells display the phenotype of stromal cells and are therefore undetectable using routine histological assessments. Our results highlight the importance of genetic analyses and the application of mutation-specific antibodies in the identification of potentially recurrent-tumor-initiating cells, which may help better predict patient survival and disease outcome.

## 1. Introduction

Melanoma is a highly aggressive malignancy, which often gives rise to metastases and local recurrences following tumor-free staging [1,2]. The histopathological examination of excisional and re-excisional samples and sentinel lymph node biopsies constitutes a fundamental part of the prognostic evaluation of this disease. Thus, the ability to detect recurrent-tumor-initiating cells in the tissue sample is highly important both for prognostic purposes and for managing the disease.

As a result of tumor heterogeneity, tumor cells often differentially express phenotypic markers [3,4], rendering their detection during routine histological assessments difficult. Epithelial-mesenchymal transition, dedifferentiation, and acquisition of stem-cell characteristics have been shown to make tumor cells more malignant [3,5,6]. Although the mechanisms by which these features are acquired have not yet been fully elucidated, epigenomic and genomic alterations are thought to be the key drivers of this malignant heterogeneity [7,8].

Cell fusion has been implicated in playing a major role in tumor progression in numerous tumor types [9,10,11]. Several studies have found that hybrid cells of tumor-stromal cell fusion are more malignant than the parental tumor cells and contribute to tumor heterogeneity [12]. Moreover, it has been shown that cell fusion might alter the phenotype of the parental cells [13,14,15] and that it can be an alternative way of epithelial mesenchymal transition [16,17,18].

Therefore, we hypothesized that tumor cells could similarly adopt a stromal phenotype by fusing with peritumoral stromal cells, while maintaining their oncogenic properties. Such fusion may lead to a cell type with mixed features that can evade immune recognition and clinical detection and therefore promote tumor recurrence. Thus, the detection of such hybrid cells in the intra- and peritumoral stroma could be significant for the accurate histopathological diagnosis and subsequent therapy.

## 2. Results

### 2.1. The BRAF^V600E^ Protein Is Expressed in Subpopulations of Peritumoral Stromal Cells

**Figure 1:**
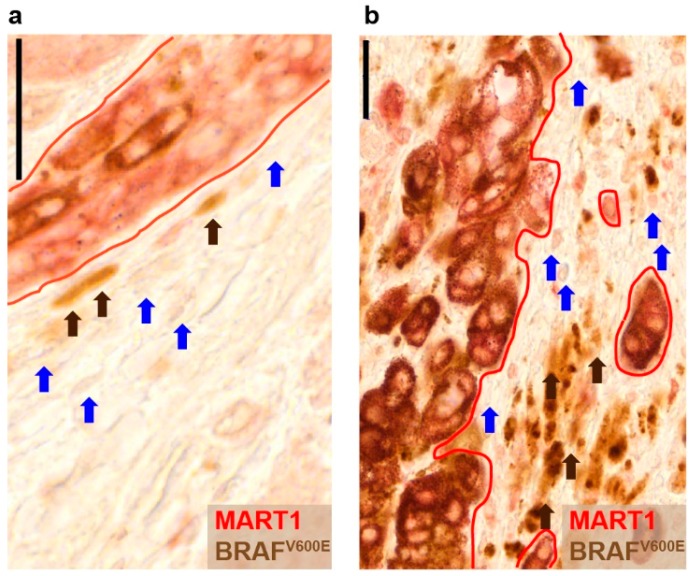
Peritumoral stromal cells express the melanoma-derived oncogenic BRAF^V600E^ protein. (**a**,**b**) Tissue samples of a patient with *BRAF^V600E^* melanoma stained for the melanoma marker melanoma antigen recognized by T-cells (MART1) (red), the BRAF^V600E^ mutant protein (brown) and hematoxylin (blue). Brown arrows indicate peritumoral MART1−/BRAF^V600E^+ cells displaying fibroblast (**a**) or macrophage (**b**) morphology. Blue arrows indicate MART1−/BRAF^V600E^− stromal cells (light blue from hematoxylin). Red lines indicate MART1+ melanoma cells. Black bars indicate 50 µm.

To identify potential recurrent tumor-initiating cells, we screened for peritumoral cells that display a stromal phenotype but carry tumor-derived oncogenic information. For this we performed dual immunohistochemical staining on *n* = 11 patient-derived tissue samples of *BRAF^V600E^* melanoma with MART1 (melanoma antigen recognized by T-cells) and BRAF^V600E^. *BRAF^V600E^* is an oncogenic somatic mutation present in approximately 50%–60% of malignant melanomas. The staining revealed that, in addition to melanoma cells, BRAF^V600E^ is also expressed in some subpopulations of MART1− peritumoral stromal cells with fibroblast and macrophage morphology (Figure 1a,b). To investigate whether the mutant protein originated from the tumor cells, we tested the stroma of *n* = 5 *BRAF^WT^* melanomas for the presence of the BRAF^V600E^ protein. However, we could not detect the mutant protein in *BRAF^WT^* melanoma tissue samples (Supplementary Materials Figure S1), suggesting that the presence of the mutation in the stromal cells could not occur without the neighboring melanoma cells carrying the mutation. Nevertheless, though the antibody has been reported to be highly specific, and we did not see any specific signals in *BRAF^WT^* melanoma tissue samples, we cannot rule out nonspecific staining especially in case of macrophages.

### 2.2. Some Peritumoral Fibroblasts and Macrophages Carry the BRAF^V600E^ Mutation in Primary Melanoma, Melanoma Metastasis and a Tumor-Free Re-Excision Sample

**Figure 2:**
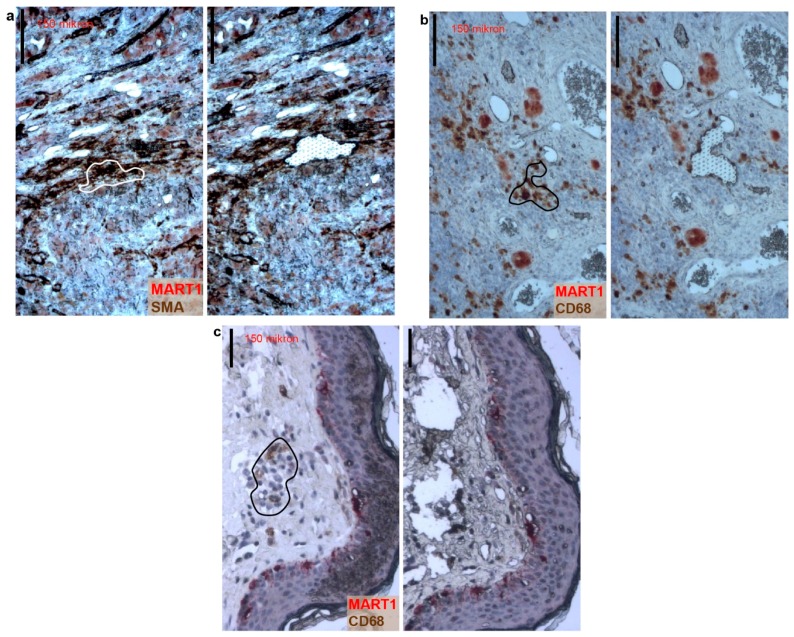
Peritumoral stromal cells contain the melanoma-derived oncogenic *BRAF^V600E^* at the genomic level. (**a**) *BRAF^V600E^* melanoma tissue sample stained for MART1 (red), smooth muscle actin (SMA) (brown), and hematoxylin (blue) before (**left** panel) and after (**right** panel) laser-capture microdissection of MART1−/SMA+ fibroblasts (white line); (**b**,**c**) Tissue samples of *BRAF^V600E^* (**b**) primary melanoma and (**c**) melanoma metastasis stained for MART1 (red), CD68 (brown), and hematoxylin (blue) before (**left** panels) and after (**right** panels) laser-capture microdissection of MART1−/CD68+ macrophages (black line). Black bars indicate 150 µm.

We wanted to confirm that the staining we observed in the *BRAF^V600E^* samples was specific, so we examined whether this oncogenic information is also present in the peritumoral cells at the genetic level. Therefore, we dual-stained *BRAF^V600E^* primary melanoma tissue samples with MART1 and either the fibroblast marker smooth muscle actin (SMA) or the monocyte-macrophage marker CD68, and isolated cell compartments consisting of 20–50, clearly MART1− but either SMA+ or CD68+ cells with fibroblast and macrophage morphology, respectively, using laser-capture microdissection (Figure 2a,b). Subsequent allele specific mutation detection PCR analyses performed on the dissected stromal cells revealed that the *BRAF^V600E^* mutant allele was present in peritumoral MART1−/SMA+ fibroblasts and MART1−/CD68+ macrophages. In addition to primary melanoma tissue samples, such peritumoral stromal cells carrying the melanoma-derived mutation were detected in lymph node and cutaneous melanoma metastases (Figure 2c). Surprisingly, *BRAF^V600E^* was also detected when analyzing MART1− macrophages dissected from a histologically tumor-free re-excision sample from a patient who subsequently developed a local recurrence. Having dissected peritumoral stromal cells from *BRAF^WT^* melanoma tissue samples, we only detected the wild-type allele, providing further evidence that *BRAF^V600E^* is only present in peritumoral cells that are adjacent to a mutant tumor. In total, we examined 86 dissected samples of 19 patients, and out of 12 patients with *BRAF^V600E^* melanoma, we found *BRAF^V600E^*-containing peritumoral cells in the tissue samples of five patients. The proportion of mutant alleles detected in these cell populations varied between 0.5% and 30% (Table 1 and Supplementary Materials Table S1).

These results indicate that some peritumoral stromal cells contain a melanoma-derived oncogene at both the DNA and protein levels. The possible recurrent tumor-initiating potential of these cells is supported by our clinical observations of the local recurrence rate in our melanoma patients diagnosed before 1998. Patients who had primary melanoma removed completely, based on a clinical diagnosis of a non-melanoma skin lesion and with histologically tumor-free excision margins developed local recurrence significantly more often (*p* = 0.7 × 10^−5^, 12 out of 228 patients) than patients with melanomas excised with 5-cm margins (8 out of 935 patients), implying that recurrent-tumor-initiating cells could indeed be present in the peritumoral stroma of malignant melanoma.

## 3. Discussion

After the complete resection of primary cutaneous melanoma, patients occasionally develop local recurrence, which is considered an independent prognostic factor [1]. This can be explained by hidden tumor-initiating cells containing tumor-derived genetic information as local minimal residual disease in the peritumoral area. In addition, such tumor-derived cells can also spread to distant areas such as lymph nodes.

Tumor cells are commonly identified during routine histological assessments using markers that are often insufficiently sensitive to distinguish tumor cells from stromal cells, which is supported by additional genetic analyses of sentinel lymph nodes [19]. The possible role of isolated tumor cells in the sentinel lymph node is debated, and the predictive value of additional diagnostic approaches, such as melanoma-marker detection at the mRNA level, to date, is inconclusive [20]. Nevertheless, some studies using enhanced pathological assessments have found a link between the presence of single tumor cells in the sentinel lymph node and worse disease outcome compared with completely tumor-free sentinel lymph nodes [21,22,23], and other improved tumor detection methods, such as quantitative immunocytochemistry combined with single-cell comparative genomic hybridization, have been found to better predict patient survival and disease outcome [24]. The incomplete removal of tumor cells often results in a higher recurrence rate: small excision margins lead to a more frequent tumor recurrence even if the excision margins are assessed to be tumor-free [2,25,26,27]. These data imply that recurrent-tumor-initiating cells with an altered phenotype could indeed be present in the peritumoral stroma of malignant melanoma, allowing them to remain undetected during routine diagnostic procedures.

In this study, we examined patient-derived melanoma tissue samples and detected peritumoral cells displaying stromal cell phenotype but carrying the oncogenic *BRAF^V600E^* mutation characteristic of the adjacent melanoma cells. First, we detected the mutated protein with a mutation-specific antibody. Even though nonspecific staining of the antibody in peritumoral mononuclear cells have been reported [28,29], the fact that peritumoral fibroblasts were also positive argues for a specific signal. Moreover, we only observed positivity in peritumoral cells adjacent to *BRAF^V600E^* melanoma. Nevertheless, to confirm the presence of the oncogene in these cells, we also performed genomic analyses and showed that the mutation is also present in cells with a stromal cell phenotype adjacent to *BRAF^V600E^* melanoma cells at the genetic level. We believe the mutation originates from tumor cells, as we did not detect the mutation in the stroma of *BRAF^WT^* melanoma samples. Possible mechanisms for the transfer of tumor-derived information to stromal cells include tumor-stromal cell fusion, exosomal protein transfer and the phagocytosis of tumor debris. However, subpopulations of peritumoral stromal cells carried the *BRAF^V600E^* mutation not only at protein but also at genomic level, suggesting cell fusion as the underlying mechanism. It is important to mention that, since melanoma cells can lose MART1 expression, especially on the periphery of the tumor, cell morphology and the expression patterns of CD68 or SMA were all taken into account during the process of peritumoral cell identification.

Cell fusion studies based on *in vitro* observations and mouse models suggest that cell fusion can confer an evolutionary benefit, such as increased metastatic potential [30] or drug resistance [31] to certain tumor–stromal cell hybrid clones. However, the majority of *in vitro* spontaneously formed hybrid cells undergo apoptosis [32], most likely as a result of mitotic stress. Therefore, it is possible that tumor stromal cell fusion is rather an antitumor mechanism, eliminating most of the hybrid tumor cells but occasionally giving rise to highly malignant tumor cell clones.

Studies in mice [33] imply that cell fusion may also take place in human tumors *in vivo*. However, it is very difficult to detect tumor–stromal cell fusion on a genetic level in humans. To address this difficulty, tumors developing in patients who have received bone marrow transplants have been investigated: genetic material originating from the donor was detected in the patients’ tumor cells [34,35], strongly suggesting a fusion event between recipient tumor cells and donor hemopoietic cells. However, inflammation resulting from treatment (e.g., whole body irradiation or chemotherapeutics) preceding bone-marrow transplantation has been reported to promote cell fusion [36,37,38]; therefore, further studies investigating fusion between tumor and stromal cells are clearly required.

Nevertheless, human *in vivo* studies demonstrating the relevance of tumor–stromal cell fusion in the clinical diagnosis or treatment of cancer have been lacking.

In conclusion, our results suggest that peritumoral stromal cells contain melanoma-specific oncogenic properties such as the *BRAF^V600E^* mutation derived from the neighboring tumor cells, potentially as a result of cell fusion. Based on the literature data, these cells, following a reversal to a tumorous phenotype [12], can possibly contribute to tumor recurrence. These cells are phenotypically indistinguishable from peritumoral stromal cells and are therefore not accurately detectable by routine histological assessments. Our results highlight the importance of genetic analysis and mutation-specific antibodies, especially in the case of histologically tumor-free tissue samples, for which improved methods for the detection of tumor cells have already been shown to better predict survival and outcome. Although yet to be confirmed in clinical trials, the use of genetic analyses and mutation-specific antibodies could have important prognostic and therapeutic consequences and could enable the detection of recurrent-tumor-initiating cells.

## 4. Experimental Section

### 4.1. Tissue Samples and Determination of BRAF Mutational Status

Tissue samples from *n* = 11 patients with *BRAF^V600E^* and *n* = 5 patients with *BRAF^WT^* melanoma were examined for BRAF^V600E^ protein expression. The corresponding patient numbers for genetic analyses were *n* = 11 in the case of *BRAF^V600E^* melanoma and *n* = 8 in the case of *BRAF^WT^* melanoma. The *BRAF* mutational status of melanomas was determined with cobas^®^ 4800 BRAFV600 Mutation Test (Roche Molecular Diagnostics, Pleasanton, CA, USA).

### 4.2. Immunohistochemistry

Formalin-fixed, paraffin-embedded tissue sections of patients with *BRAF^V600E^* malignant melanoma were stained with rabbit monoclonal MART1 (clone A19-P, DB Biotech, Kosice, Slovakia), mouse monoclonal SMA (clone 1A4, DAKO, Glostrup, Denmark), CD68 (clone PG-M1, DAKO), and BRAF^V600E^ (Clone VE1, Springbio, Pleasanton, CA, USA) primary antibodies. The Bond Polymer Refine Detection Kit and the ChromoPlex 1 Dual Detection Kit (both from Leica, Wetzlar, Germany) were used for the visualization of single and dual histochemical staining, respectively, according to the manufacturer’s instructions. All immunohistochemical staining was scanned with a Pannoramic MIDI Slide Scanner (3DHISTECH Ltd., Budapest, Hungary) and analyzed with Pannoramic Viewer software (3DHISTECH Ltd.).

### 4.3. Laser-Capture Microdissection and Detection of the BRAF^V600E^ Allele

30–100 cells were dissected from tissue sections stained either with MART1 and SMA or with MART1 and CD68 using a Palm Microbeam (Carl Zeiss Microscopy, Jena, Germany). Samples were digested with proteinase K (QIAGEN, Venlo, The Netherlands). Mutant allele (BRAF_476_mu) and gene reference (Braf_rf) TaqMan^®^ Mutation Detection Assays (Life Technologies, Carlsbad, CA, USA) were used to detect the *BRAF^V600E^* mutant allele in the dissected samples. Samples with Δ*C*_t_ (*C*_tmut_ − *C*_tref_) < 8 and *C*_tmut_ < 40 were considered carrying the mutant allele. MART1+ tumor cells dissected from *BRAF^WT^* and *BRAF^V600E^* melanoma were used as negative and positive controls, respectively.

### 4.4. Statistical Analysis

Local recurrence rate in melanoma patients were compared with the Fisher’s exact test. The level of significance was set to 0.05.

## Figures and Tables

**Table 1:** Peritumoral fibroblasts and macrophages in *BRAF^V600E^* melanoma carry the melanoma-derived *BRAF^V600E^* mutation at the genetic level.

Tissue ^1^	*N*_pos_/*N*_ex_^2,3^	
	Fibroblasts ^4^ Dissected	Macrophages ^5^ Dissected
*BRAF^V600E^* primary melanoma	2/2	1/1
*BRAF^V600E^* melanoma metastasis	2/4	2/4
Histologically tumor-free tissue (from patients with *BRAF^V600E^* melanoma)	0/4	1/3
*BRAF^WT^* primary melanoma	0/4	0/1

^1^ Tissue samples were dual-stained either with melanoma antigen recognized by T-cells (MART1) & smooth muscle actin (SMA) or with MART1 & CD68 and examined by a certified pathologist (IBN); ^2^ *N*_pos_: number of patients where *BRAF^V600E^*-positive peritumoral stromal cells were found; *N*_ex_: the number of patients examined; ^3^ Dissected cell samples were considered positive for *BRAF^V600E^* if the prevalence of the mutant allele was higher than 0.39% (Δ*C*_t_ < 8); ^4^ MART1−/SMA+ cells were considered fibroblasts; ^5^ MART1−/CD68+ cells were considered macrophages.

## Supplementary Materials

Supplementary materials can be found at http://www.mdpi.com/1422-0067/17/6/980/s1.

## Abbreviations

MART1	melanoma antigen recognized by T-cells
SMA	smooth muscle actin
