# Supplementary material for: Melanoma-Derived BRAFV600E Mutation in Peritumoral Stromal Cells: Implications for in Vivo Cell Fusion
Source: Int J Mol Sci. 2016 Jun 21;17(6):980. doi: 10.3390/ijms17060980 (PMC4926511; doi:10.3390/ijms17060980)
Supplement: Supplementary file 1 [file ijms-17-00980-s001.zip › ijms-127525-Supplementary Materials/ijms-127525-supplementary-Figure S1.pdf]

Supplementary Materials: Melanoma-Derived *BRAF*^{V600E} Mutation in Peritumoral Stromal Cells: Implications for *in Vivo* Cell Fusion

Zsuzsanna Kurgyis, Lajos V. Kemény, Tünde Buknicz, Gergely Groma, Judit Oláh, Ádám Jakab, Hilda Polyánka, Kurt Zänker, Thomas Dittmar, Lajos Kemény and István B. Németh

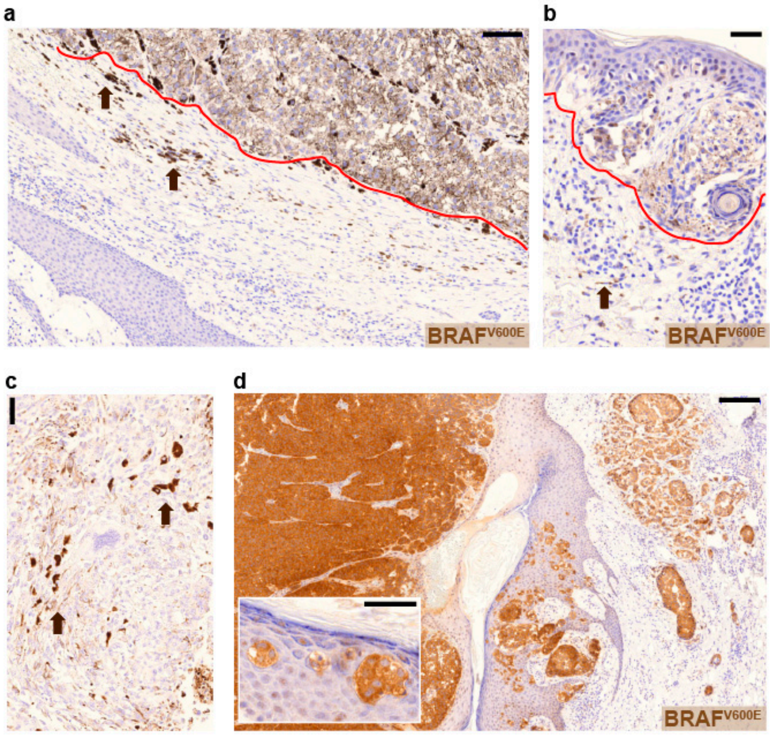

Figure S1. BRAF^{V600E} is not expressed in peritumoral stromal cells BARF^{WT} melanoma. (**a**,**b**) BARF^{WT} melanoma tissue sample stained with BRAF^{V600E} (light brown) and hematoxylin (blue) (**c**) *BRAF*^{WT} melanoma tissue sample stained without primary antibody; (**d**) *BRAF*^{V600E} melanoma tissue sample stained with BRAF^{V600E} (light brown) and hematoxylin (blue). Red lines demarcate tumor cells and brown arrows (**a**–**c**) indicate dark brown granules that correspond to melanin pigments. Scale bars indicate 100 µm (**a**); 50 µm (**b**, **c** and **d** small panel) and 200 µm (**d** large panel).
